# Safety of AFM11 in the treatment of patients with B-cell malignancies: findings from two phase 1 studies

**DOI:** 10.1186/s13063-022-06982-7

**Published:** 2023-01-03

**Authors:** Max Topp, Monika Dlugosz-Danecka, Aleksander B. Skotnicki, Galina Salogub, Andreas Viardot, Andreas K. Klein, Georg Hess, Christian S. Michel, Sebastian Grosicki, Alex Gural, Sylvia E. Schwarz, Kerstin Pietzko, Ulrike Gärtner, András Strassz, Leila Alland, Jiri Mayer

**Affiliations:** 1 Universitätsklinikum Würzburg, Würzburg, Germany; 2 Maria Sklodowska-Curie National Research Institute of Oncology, Kraków, Poland; 3 Department of Haematology, Jagiellonian University, Kraków, Poland; 4 Almazova National Medical Research Center, St Petersburg, Russia; 5 Klinik Für Innere Medizin III, Universitätsklinikum Ulm, Ulm, Germany; 6 Tufts Medical Center, Boston, MA USA; 7 Universitätsmedizin Mainz, Mainz, Germany; 8 Department of Hematology and Cancer Prevention, Faculty of Health Sciences, Medical University of Silesia, Katowice, Poland; 9 Hadassah Medical Center, Jerusalem, Israel; 10 Affimed GmbH, Heidelberg, Germany; 11 University Hospital Brno, and Masaryk University, Brno, Czech Republic

**Keywords:** Non-Hodgkin lymphoma, Acute lymphoblastic leukaemia, AFM11, Neurotoxicity, T-cell engager

## Abstract

**Background:**

The prognosis for patients with relapsed and/or refractory (R/R) non-Hodgkin’s lymphoma (NHL) or acute lymphoblastic leukaemia (ALL) remains poor, with existing treatments having significant side effects. Developed for the treatment of these cancers, AFM11 is a tetravalent, bispecific humanised recombinant antibody construct (TandAb®) designed to bind to human CD19 and CD3 and lead to the activation of T cells inducing apoptosis and killing of malignant B cells.

**Methods:**

Two open-label, multicentre, dose-escalation phase 1 studies evaluated the safety, pharmacokinetics and activity of AFM11 in patients with R/R CD19-positive B cell NHL (AFM11-101) and in patients with CD19 + B-precursor Philadelphia-chromosome negative ALL (AFM11-102). Adverse events (AEs) were assessed and recorded; imaging (NHL) or bone marrow assessment (ALL) were used to evaluate response. Additional pharmacodynamic assays undertaken included cytokine release analysis and B-cell and T-cell depletion.

**Results:**

In AFM11-101, 16 patients with R/R NHL received AFM11 in five different dose cohorts. Of which, 14 experienced drug-related treatment-emergent AEs (TEAEs) [including five serious AEs (SAEs)], five patients experienced dose-limiting toxicity (DLT) and ten patients discontinued the study. The high number of neurological events led to a decrease in infusion frequency during the study. No objective response to treatment was observed. In AFM11-102, 17 patients with R/R ALL received AFM11 in six different dose cohorts. Thirteen patients experienced drug-related TEAEs (including four SAEs), DLTs occurred in two patients and five patients discontinued the study. An objective response was recorded in three patients. The maximum tolerated dose could not be determined in either study due to early termination.

**Conclusions:**

AFM11 treatment was associated with frequent neurological adverse reactions that were severe in some patients. In ALL, some signs of activity, albeit short-lived, were observed whereas no activity was observed in patients with NHL; therefore, further clinical development was terminated.

**Trial registration:**

NCT02106091. Safety Study to Assess AFM11 in Patients With Relapsed and/or Refractory CD19 Positive B-cell NHL. Registered April 2014. NCT02848911. Safety Study to Assess AFM11 in Patients With Relapsed or Refractory Adult B-precursor ALL. Registered July 2016.

**Supplementary Information:**

The online version contains supplementary material available at 10.1186/s13063-022-06982-7.

## Background

Non-Hodgkin’s lymphoma (NHL) comprises a large group of lymphoproliferative malignancies that originate either from malignant B or T lymphocytes. NHL was the seventh most common cancer diagnosis in the USA in 2020 and the most common aggressive subtype is diffuse large B-cell lymphoma (DLBCL), which accounts for approximately 25–45% of all NHL cases worldwide [1].

While patients with indolent NHLs have relatively good prognoses with long median survival rates, even in cases of relapsed disease, for patients with aggressive lymphomas, who relapse or are refractory to first-line treatment, the prognosis is poor, despite multiple treatment options [2].

Acute lymphoblastic leukaemia (ALL) is an aggressive type of leukaemia characterised by an overproduction of lymphoblasts in the bone marrow and the peripheral blood. It can spread to the lymph nodes, spleen, liver and central nervous system and if untreated can be rapidly fatal. ALL occurs in both adults and children; in adults, it is a rare disease (7–8% of all types of leukaemia), with 5-year survival rates of 31.4% in those aged 40–64 years and 19.8% in those aged 65–74 years [3]. Historically, the prognosis for patients with relapsed and/or refractory (R/R) ALL has been extremely poor, with median overall survival of around 6 months and complete remission rates of 20–40% despite intensive salvage chemotherapy and haematopoietic stem cell transplantation [4, 5]. However, the introduction of novel therapies has improved overall and relapse-free survival [6]. The CD19 antigen, a type I transmembrane glycoprotein belonging to the immunoglobulin Ig superfamily, is specifically expressed in normal and neoplastic B-cells and has been shown to accelerate B-cell lymphomagenesis [7, 8]. The CD19 antigen is expressed on the B cell membrane from early B cell development through differentiation into plasma cells [9]. The expression of CD19 at various developmental stages of B cells makes it an ideal target to treat B cell-associated malignancies. CD19 expression has also been demonstrated to play an active role in driving cancer growth by stabilising the concentration of the MYC oncoprotein [10]. Previously, anti-CD19 antibody drug conjugates such as coltuximab ravtansine (SAR3419) demonstrated promising antitumour activity with acceptable safety profile in NHL [11]; however, the phase 2 study was discontinued prematurely due to the low clinical response rates observed in patients [12]. Since then blinatumomab, a CD3/CD19 bispecific T-cell engager has been licensed for the treatment of Philadelphia-chromosome negative and Philadelphia-chromosome positive R/R B-precursor ALL [13].

In a phase 3 clinical trial in R/R disease, blinatumomab was significantly more effective than standard salvage chemotherapy; however, neurotoxicity and cytokine release syndrome were frequent side effects [14]. Its short half-life also necessitated continuous infusion for 4 weeks of every 6-week cycle. Consequently, there remains a need for effective agents with fewer side effects.

AFM11 is a tetravalent, bispecific humanised recombinant antibody construct (TandAb®) developed for the treatment of CD19-positive NHL and ALL [9]. AFM11 was designed to bind to human CD19 and CD3 and forms an ‘immunological synapse’ leading to the activation of T cells inducing apoptosis and killing of malignant B cells [9]. AFM11 has two binding sites for CD19 and CD3 antigens each [9] and in vitro studies showed AFM11 to have an affinity for CD3 approximately 100 times that of blinatumomab suggesting the potential for greater clinical efficacy [9]. In addition, the greater molecular weight of AFM11 prevents glomerular filtration and may also allow a less burdensome infusion schedule. In vivo anti-tumour activity of AFM11 was investigated in a Raji tumour xenograft model in NOD/SCID mice reconstituted with human peripheral blood mononuclear cells. All animals in the highest dose group achieved complete tumour regression [9].

Here, we present data from two discontinued phase 1 studies that evaluated the safety, pharmacokinetics (PK) and activity of AFM11 in patients with R/R CD19-positive B cell NHL (AFM11-101 [NCT02106091]) and in patients with CD19 + B-precursor Philadelphia-chromosome negative ALL (AFM11-102 [NCT02848911]).

## Methods

### AFM11-101

AFM11-101 was a phase 1 open-label, first-in-human, dose-escalation study conducted at 10 sites in Germany, Czech Republic, Poland and the USA between October 2014 and September 2018. The primary objective of the study was to determine the safety and tolerability of AFM11 administered intravenously at weekly intervals over a period of 4 weeks (1 cycle) in patients with NHL, investigating escalating doses and different infusion durations. Secondary objectives included determination of the maximum tolerated dose (MTD; highest dose at which < 33% of patients experienced a dose-limiting toxicity [DLT; any grade 3 or higher non-haematological toxicity or grade 4 or higher haematological toxicity or any treatment delay ≥ 21 days because of drug-related adverse events (AEs)]), with the overall aim of identifying the dose and infusion time for a phase 2 study. In addition, the PK and the biological activity of AFM11 as well as pharmacodynamic (PD) markers in blood were assessed.

#### Study participants

This study included male or female patients ≥ 18 years with indolent or aggressive CD19 + NHL, who had relapsed or were refractory to standard therapy, which must have included treatment with rituximab plus chemotherapy, and were not eligible for a curative treatment option. All patients must have had measurable disease (at least one lesion ≥ 1.5 cm) documented by a computed tomography (CT) scan, an Eastern Cooperative Oncology Group (ECOG) performance status of ≤ 2, and a life expectancy of ≥ 6 months.

Patients were excluded if their total number of B cells (healthy and malignant combined) in peripheral blood exceeded the upper limit of normal in healthy individuals (assessed by flow cytometry) or if they had central nervous system (CNS) involvement, a history of malignancy other than B-cell lymphoma within 5 years before study entry, active autoimmune disease requiring immunosuppression or uncontrolled infection. Patients who received an autologous haematopoietic stem cell transplant or treatment with alemtuzumab within 12 weeks of the start of AFM11 treatment, chemotherapy, radiotherapy, therapy with an antibody, an investigational drug or corticosteroid treatment within 4 weeks of the start of AFM11 treatment, or who had prior treatment with a CD19-targeting T-cell engager, including CD19 CAR-T cells, were also excluded.

A total of 40 patients were anticipated to be enrolled based on the number of dose cohorts; the actual number of enrolled patients was dependent upon the number of dose levels assessed and the number of DLTs observed at each dose level.

#### Study design

The study used an open-label, dose-escalating design, with the starting dose selected based on the minimum anticipated biological effect level determined from in vitro data and PK models as no relevant in vivo data were available. AFM11 was planned to be escalated at the predefined doses of: 0.0003, 0.001, 0.003, 0.01, 0.03, 0.09, 0.20, 0.40, 0.80, 1.5 and 2.5 μg/kg. AFM11 was given as an intravenous infusion over 4, 24, or 48 h in a stepwise manner with a separate dose escalation scheme for each infusion time until an MTD had been reached (Supplementary Fig. 1). For the initial 4-h infusion step, an accelerated titration design [15] with 1 patient per cohort was used until a DLT or activity (B-cell depletion or an increase in interleukin-2 [IL-2], IL-6 or interferon-γ [IFN-γ]) was observed, at which point a 3 + 3 escalation method with 3–6 patients per cohort was used. If a DLT occurred during the accelerated titration phase at dose levels of 0.0003, 0.001, 0.003 or 0.01 μg/kg, the trial immediately transitioned to the next infusion time.

Escalation to the next dose cohort only occurred when patients receiving the prior dose level had completed cycle 1 (4 weeks). Patients received the prior cohort dose in cycle 1 week 1 and escalated to their cohort dose in cycle 1 weeks 2–4. Intrapatient dose reduction was only permitted if a patient experienced cytokine release syndrome or neurotoxicity; intrapatient dose escalation only occurred between cycle 1 week 1 and cycle 1 week 2, and dose escalation beyond the assigned cohort dose was not permitted. Dosing could be delayed by up to 21 days in each cycle to allow toxicity to return to grade 1 or baseline. If a dose could not be given within 21 days of the scheduled date, the patient was withdrawn from the study.

Initially, a dosing frequency of 5 times/week in week 1 and 3 times/week in weeks 2–4 was selected based on animal models that demonstrated more intensive dosing produced the most effective inhibition of tumour growth. After the first five patients were treated, dosing frequency was reduced to once weekly as this was expected to result in a more favourable safety profile.

Patients were hospitalised for at least 24 h or the duration of the infusion. At PK sampling visits, patients were hospitalised for 24, 48, or 72 h for the 4-, 24- and 48-h infusions, respectively. Patients who were clinically stable or showed either B-cell depletion or T-cell expansion after cycle 1 and who had no unacceptable toxicity could receive a second cycle of therapy at the investigator’s discretion. Based on either the investigator’s decision or the patient’s request, patients could be withdrawn from the study at any time.

#### Additional medication

After the first 5 patients had been treated, the protocol was amended to include mandatory premedication with dexamethasone 20 mg (or equivalent) and antihistamines 1 h prior to the first and second infusions of AFM11 in cycle 1 and with dexamethasone 10 mg (or equivalent) and antihistamines 1 h before all subsequent infusions. Any other medication necessary for the patient’s wellbeing was given at the discretion of the investigator.

#### Assessments

##### Primary objective

AEs were graded according to Common Terminology Criteria for Adverse Events (CTCAE) version 4.03, with assessments including clinical examinations, the assessment of AEs (especially DLT), and laboratory parameters. AEs were captured from the time of consent until 4 ± 1 weeks from the end of the last infusion of AFM11. The investigator assessed whether the AE was not/unlikely related, possibly related, probably related or definitely related to AFM11. Serious AEs (SAEs) were defined as an untoward medical occurrence that resulted in death or persistent or significant disability/incapacity, was life-threatening, required hospitalisation or was a congenital anomaly or birth defect, or an important medical event as decided by medical and scientific judgement.

Tumour imaging by fluorodeoxyglucose-positron emission tomography and CT was conducted at screening and at 2 (± 1) weeks after the end of cycle 1. Responses were determined by the investigator in accordance with Cheson criteria [16]. In patients with bone marrow involvement at baseline, a bone marrow biopsy was required to confirm a complete response.

Antidrug antibodies (ADAs) were measured by an enzyme-linked immunosorbent assay before the first dose and at the Tumour Assessment Visit.

##### Secondary objectives

Blood samples for determination of AFM11 serum concentration were drawn pre-dose and at regular intervals after the start of the infusion until 72, 72 and 48 h after the end of the 4-, 24- and 48-h infusions, respectively, in weeks 1 and 4 of cycle 1.

Pharmacodynamic analysis included a modified standard panel for lymphocyte analysis by flow cytometry to characterise the basic lymphocyte subset (absolute and relative CD3 + , CD4 + , CD8 + T cells and the CD4:CD8 ratio, as well as CD20 + B cells). Inclusion of absolute and relative CD16 + /CD56 + and CD3 − natural killer (NK) cells was optional. In Germany, and optionally in other centres, the expression of activation and memory markers on T cell subsets was assessed by flow cytometry.

Other pharmacodynamic assays included systemic cytokine release, assessed by multiplex analysis of a panel of proinflammatory cytokines*,* at pre-dose, during infusion and post-dose, quantification of the cytolytic potential of T cells pre- and post-dose, and exploratory biomarker analysis pre- and post-dose with an additional sample to be taken in case of a ≥ grade 3 neurological event.

The safety population included all patients who had received at least one dose of AFM11. The dose determination set comprised all patients who had received at least 4 weeks of treatment or had stopped treatment for reasons of toxicity. The DLT analysis set included all patients in the dose determination set. The PK population was defined as all patients who received at least one dose of AFM11 and for whom serum concentrations of AFM11 were measured.

#### Statistical analysis

There was no formal statistical analysis in this study; results are presented using descriptive statistics for continuous variables that include the number of patients, arithmetic mean, SD, median, minimum and maximum. Summary statistics for categorical variables contain count and percentage based on the number of patients in a cohort and the selected analysis population.

### AFM11-102

AFM11-102 was a phase 1, multicentre, open-label dose escalation study of AFM11 in patients with R/R CD19 + adult B-precursor ALL conducted at 12 sites in five countries (Austria, Czech Republic, Israel, Poland and Russia) between October 2016 and May 2019. The primary objectives were to determine the MTD (the highest dose at which < 33% of patients experienced a DLT defined as any grade 3 or higher non-haematological toxicity or grade 4 or higher haematological toxicity or any treatment delay ≥ 7 days because of drug-related adverse events AEs) and to evaluate the safety and tolerability of increasing doses and different infusion times of AFM11 in patients with ALL. Secondary objectives included assessment of the PK and antitumour activity of AFM11 after at least 1 cycle of therapy. In addition, the biological activity and PD markers in blood were assessed.

#### Study participants

The study included men or women aged ≥ 18 years with a diagnosis of R/R CD19 + B-precursor Philadelphia-chromosome negative ALL who were not candidates for bone marrow transplantation with curative intent. CD19 expression must have been confirmed by either staining or flow cytometry of a recent bone marrow biopsy. Patients were required to have failed or be intolerant to therapy with at least two tyrosine kinase inhibitors, > 5% blasts in their bone marrow, an ECOG performance status of ≤ 2 and a life expectancy of ≥ 3 months.

Patients were excluded from the study if they had received autologous or allogeneic haematopoietic stem cell transplant within the previous 3 months; had active graft versus host disease; had received prior treatment with blinatumomab or other CD19 targeting T-cell engager; had been treated with a donor lymphocyte infusion, cancer chemotherapy, an antibody or antibody construct or any investigational agent; or had received regular corticosteroids or other immunosuppressive in the 4 weeks prior to study entry. Patients with CNS involvement, a history of CNS pathology, abnormal renal or hepatic function, history of malignancy other than ALL, uncontrolled infection, clinically relevant coronary artery disease or other relevant disease were also excluded.

A total of 50 patients were estimated to be enrolled based on the number of dose cohorts; the actual number of enrolled patients was dependent upon the number of dose levels assessed and the number of DLTs observed at each dose level.

#### Study design

The study was an open-label, dose-escalation study that followed a modified accelerated-titration design until DLT was observed or two patients exhibited grade 2 non-haematological toxicity related to the study drug during cycle 1 [15]. AFM11 was given as a continuous infusion over 2 weeks, with a dose titration step from week 1 to week 2 (Supplementary Fig. 2). This schedule followed the same approach as established for blinatumomab in ALL where it has been shown to reduce the risk of cytokine release syndrome and neurotoxicity [17, 18]. The starting dose of AFM11 in cohort 1 was 0.0007 μg/kg/week for week 1 increasing to 0.002 μg/kg/week for week 2; the starting dose was selected as it is below the EC10 of AFM11 and below the weekly dose used in AFM11-101, and the cumulative weekly dose in both studies is similar. Dose escalation followed a modified Fibonacci regimen, with escalation of a half-log from the initial dose through cohort 4 and reduced increments (100%, 67%, 50%, 30–35%) from cohort 5.

#### Statistical analysis

There was no formal statistical analysis in this study; results are presented using descriptive statistics for continuous variables that include the number of patients, arithmetic mean, standard deviation (SD), median, minimum and maximum. Summary statistics for categorical variables contain count and percentage based on the number of patients in a cohort and the selected analysis population. 

#### Concomitant medication

In patients with a high tumour burden (e.g. more than 50% blasts, or more than 15,000 blasts/μL blood, or elevated lactate dehydrogenase more than twice the upper limit of normal), pre-treatment with 10 mg/m^2^ dexamethasone and 200 mg cyclophosphamide for up to 5 days was permitted. No immunosuppressive agents were allowed 4 weeks prior to and during AFM11 therapy; no other investigational agents were permitted during the study. Any medication necessary for the patient’s safety and well-being was given at the discretion of the investigator. Non-steroidal anti-inflammatory drugs were permitted for a fever of ≥ 37.5 °C, with the addition of dexamethasone for a fever of ≥ 38.5 °C.

#### Assessments

##### Primary objective

Safety was assessed by clinical review of all relevant parameters, including AEs, clinical laboratory evaluations (chemistry, haematology, coagulation analysis, urinalysis and detection of tumour lysis syndrome), vital signs, physical examination (including ECOG performance status), cardiac monitoring and neurological assessments. The number of patients with DLTs was summarised by severity, relatedness and preferred term/system organ class.

##### Secondary objectives

Blood samples were taken for PK analysis, biomarker and FACS analysis at regular intervals after the start of the infusion in both weeks 1 and 2. Blood samples were analysed by flow cytometry for peripheral B-cell and T-cell depletion. Serum biomarker assessments were performed at a central laboratory. Immunogenicity was assessed by analysis of the presence of ADAs using an enzyme-linked immunosorbent assay.

Efficacy was determined using bone marrow assessment performed at baseline and during evaluation visits during each cycle (days 15–18). Minimal residual disease diagnostic tests were performed at baseline and at each evaluation visit by a central laboratory.

The safety population included all patients receiving at least one dose of AFM11. The DLT analysis set included all patients who received ≥ 80% of the assigned dose and completed the DLT observation period or discontinued due to a DLT during the DLT observation period. The PK population was defined as all patients who received at least one dose of AFM11 and for whom serum concentrations of AFM11 were measured.

## Results

### AFM11-101

**Fig. 1:**
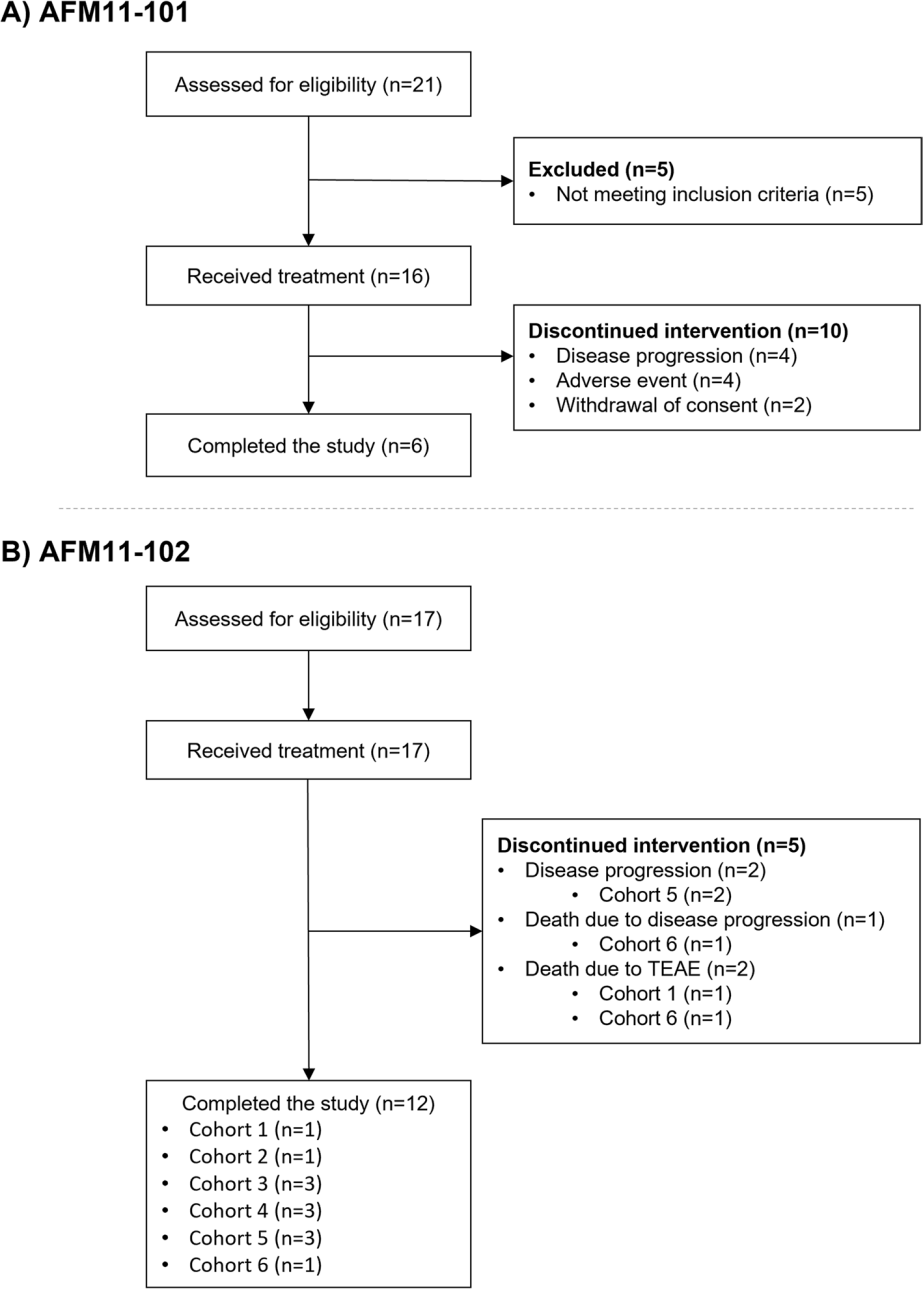
CONSORT flow diagram. Dose of AFM11 by cohort (cycle 1 week 1, cycle 1 week 2 onward) was as follows: cohort 1, 0.0007 μg/kg/week, 0.002 μg/kg/week; cohort 2, 0.002 μg/kg/week, 0.006 μg/kg/week; cohort 3, 0.007 μg/kg/week, 0.02 μg/kg/week; cohort 4, 0.02 μg/kg/week, 0.06 μg/kg/week; cohort 5, 0.06 μg/kg/week, 0.18 μg/kg/week; cohort 6, 0.13 μg/kg/week, 0.4 μg/kg/week

A total of 21 patients were enrolled, of whom 16 received at least one dose of AFM11 and were included in the safety population (Fig. 1A). The PK population could not be determined in this study because AFM11 could not be detected in the majority of PK samples; therefore, no PK data are presented.

Patient characteristics are shown in Table 1 (characteristics per cohort are provided in Table S1). Briefly, the safety population included ten men and six women with a mean (SD) age of 56.5 (16.52) years. Time since diagnosis varied between 0.9 and 15.5 years. All patients had NHL, with DLBCL as the most frequent subtype (43.8%). Most patients (75.0%) had stage IV cancer at the time of screening. All patients had received previous systemic anticancer treatment; 43.8% had received > 5 prior lines of therapy. In total, 10 patients (62.5%) were relapsed and six patients were refractory (37.5%).

**Table 1:** Patient demographics and baseline characteristics in AFM11-101

	Safety population (*n* = 16)
Age (y), mean (SD)	56.5 (16.52)
Male, *n* (%)	10 (62.5)
Diagnosis NHL, *n (%)*	16 (100)
Time since diagnosis (y), mean (SD)	4.80 (4.35)
Disease status at screening, *n (%)*	
Stage I	1 (6.3)
Stage II	1 (6.3)
Stage III	2 (12.5)
Stage IV	12 (75)
Previous treatments, *n (%)*	
At least 1 line of prior systemic therapy	16 (100)
Chemotherapy alone	15 (93.8)
Other^a^	9 (56.3)
ASCT	7 (43.8)
Anti-CD20 therapy	6 (37.5)
Targeted small molecule kinase	4 (25.0)
Immunotherapy^b^	3 (18.8)
≥ 1 prior radiotherapy	5 (31.3)

*ASCT* Autologous stem cell transplant, *NHL* Non-Hodgkin’s lymphoma, *SD* Standard deviation

^a^Other lines include R-CHOP (rituximab, cyclophosphamide, doxorubicin, vincristine, prednisolone)

^b^Immunotherapy included blinatumomab, rituximab-bendamustine, rituximab-bendamustine or rituximab-pinxantrone

These patients were assigned to five dose levels but the infusion duration used varied: 0.0003 μg/kg (*n* = 1), 0.001 μg/kg (*n* = 8), 0.003 μg/kg (*n* = 1) infused over 4 h; 0.003 μg/kg (*n* = 1) infused over 24 h; 0.003 μg/kg (*n* = 1), 0.01 μg/kg (*n* = 2), and 0.03 μg/kg (*n* = 2) infused over 48 h. Six (37.5%) patients completed cycle 1 and therefore completed the study. Of these, three (18.8%) patients continued in the study and completed an additional cycle (cycle 2). Ten patients withdrew from the study in cycle 1 because of disease progression, AEs or withdrawal of consent.

#### Safety

Treatment-emergent AEs (TEAEs) were reported in 15/16 (93.8%) patients; of these, 14 patients experienced TEAEs considered related to study treatment. The most frequent treatment-related TEAEs were pyrexia (eight patients), lymphocyte count decreased and tremor (four patients each); no dose-related trends were observed. Eight patients experienced 22 TEAEs of grade 3 or above. Of these, 11 TEAEs in five patients were considered related to study treatment (nine grade 3 and two grade 4); eight events occurred in patients in cohort 5 (Table 2).

**Table 2:** Treatment-related TEAEs with grade ≥ 3 in AFM11-101

Adverse event	AFM11 dose level (μg/kg)							
	0.0003^a^ *n* = 1	0.001^a^ *n* = 8	0.003^a^ *n* = 1	0.003^b^ *n* = 1	0.003^c^ *n* = 1	0.01^c^ *n* = 2	0.03^c^ *n* = 2	Total *n* = 16
Patients with at least 1 TEAE grade ≥ 3	0	1	1	1	0	0	2	5
Cognitive disorder	0	0	1	0	0	0	0	1
Depressed level of consciousness	0	0	0	0	0	0	1	1
Encephalopathy	0	1	0	0	0	0	0	1
Neurotoxicity	0	0	0	0	0	0	1	1
Seizure	0	0	0	0	0	0	1	1
Respiratory failure	0	0	0	0	0	0	2	2
Lymphopenia	0	1	0	0	0	0	0	1
Tachycardia	0	0	0	1	0	0	0	1
Hypotension	0	0	0	0	0	0	1	1
Confusional state	0	0	0	1	0	0	0	1

*TEAE* treatment-emergent adverse event

^a^Infused over 4 h

^b^Infused over 24 h

^c^Infused over 48 h

Six patients reported 11 SAEs. Of these, five events in five patients were considered related to study treatment; four events were nervous system disorders, and one event was a psychiatric disorder. There was one death on the study, a grade 5 event described as worsening of general condition that was not considered related to treatment. The patient had an abnormal electrocardiogram at their pre-dose assessment and a severe TEAE of tachycardia, considered possibly related to study treatment, was reported 2 days prior to and was ongoing at the time of death. All other SAEs resolved by the end of the study.

Four patients experienced TEAEs that led to withdrawal (grade 1 aphasia, grade 3 paroxysmal atrial tachycardia, grade 4 depressed level of consciousness, grade 4 neurotoxicity). The event of tachycardia was not considered related to the study treatment.

Out of 16 patients, five (31.3%) patients experienced at least one DLT (encephalopathy, cognitive disorder, confusional state, depressed level of consciousness and neurotoxicity). All DLTs occurred within the first 4 weeks of treatment and all resolved (one event with sequelae [grade 2 memory impairment following depressed level of consciousness]) between 1 and 3 days after the event (three of five patients required treatment). The events of confusional state and neurotoxicity resulted in a dose reduction and the event of depressed level of consciousness resulted in discontinuation of study treatment; there was no change to study treatment with the other two DLTs. The high number of neurological events led to the decrease in infusion frequency from 5 times/week in week 1 and 3 times/week in weeks 2–4 to once weekly for weeks 1–4.

No MTD could be determined, because there were no cohorts containing six patients on the same dose and schedule. Two DLTs occurred in cohort 5, indicating the MTD had been exceeded. In view of the high level of neurotoxicity and absence of response (see below), the study was terminated.

#### Efficacy

None of the 16 patients experienced an objective response; however, based on the haematological response criteria, six patients showed stable disease, with a best percentage change from baseline ranging from + 30 to − 50%. These results gave a disease control rate of 37.5% (95%CI 15.2–64.6%).

#### Pharmacodynamics and Immunogenicity

Total B-cell depletion (CD20 + B-cell level > 0 at baseline and 0 post baseline) occurred in three patients with baseline B cell counts of 20.0, 2.1 and 0.5, respectively.

The T-cell activity assessment was not performed due to the termination of the AFM11 clinical programme. No cytokine results were reported. No ADA results were reported, so immunogenicity could not be determined.

### AFM11-102

A total of 17 patients were enrolled and received study treatment and were included in the Safety Population (Fig. 1B). Patient characteristics are shown in Table 3 (characteristics per cohort are provided in Table S2). The safety population included 10 (58.8%) women and seven (41.2%) men, with a mean (SD) age of 46.8 (18.80) years. Mean (SD) time since diagnosis was 1.62 (1.11) years. Sixteen patients had CD19 + B-precursor Philadelphia-chromosome negative ALL and one patient had CD19 + B-precursor Philadelphia-chromosome positive ALL. All 17 patients had relapse of ALL, with a mean (SD) of 1.6 (0.70) relapses, and four patients (23.5%) had refractory disease. Four (23.5%) patients had received 1–3 prior therapy regimens and 13 (76.5%) patients had received ≥ 4 prior therapy regimens.

**Table 3:** Patient demographics and baseline characteristics in AFM11-102

	Safety population (*n* = 17)
Age (y), mean (SD)	46.8 (18.80)
Male, *n* (%)	7 (41.2)
Type of ALL, *n* (%)	
CD19 + B-precursor Philadelphia-chromosome negative ALL	16 (94.1)
CD19 + B-precursor Philadelphia-chromosome positive ALL	1 (5.9)
Time since diagnosis (y), mean (SD)	1.62 (1.11)
Total number of relapses, mean (SD)	1.6 (0.70)
Previous treatments, *n (%)*	
Chemotherapy	17 (100)
Targeted therapy^a^	3 (17.6)
Radiotherapy	3 (17.6)
Peripheral blood stem cell transplant	5 (29.4)
Time from last treatment, *n (%)*	
< 1 month	6 (35.3)
1 to < 3 months	4 (23.5)
3 to < 6 months	1 (5.9)
6 to < 12 months	2 (11.8)
≥ 12 months	4 (23.5)

*ALL* acute lymphoblastic leukaemia; *SD* standard deviation

^a^Targeted therapy included velcade asparaginase, imatinib ponatinib or rituximab

Patients were assigned to one of six cohorts. A total of 12 patients completed the study (completed cycle 1 and the final visit) and five discontinued (three died and two patients discontinued due to progressive disease; Fig. 1B). Fifteen patients were included in the DLT analysis set (two patients were excluded as they did not fulfil the criteria).

#### Safety

Overall, 17 patients participated in cycle 1, five in cycle 2 and two in cycle 3. Of these, 13 of 17 patients received ≥ 80% of the planned dose in cycle 1. The mean (SD) treatment duration was 21.59 (14.4) days.

DLTs occurred in two patients, both in cohort 6 (grade 3 cognitive disorder and grade 4 respiratory arrest). The MTD could not be determined as the study was terminated early.

A total of 16 (94.1%) patients experienced ≥ 1 TEAE. Drug-related TEAEs occurred in 13 (76.5%) patients and four patients experienced drug-related SAEs. The most frequent drug-related TEAEs were pyrexia (seven patients), alanine aminotransferase (ALT) increased, aspartate aminotransferase (AST) increased and tremor (three patients each; Table 4). Six (35.3%) patients experienced a ≥ grade 3 drug-related TEAE; the most common of which were febrile neutropenia, neutropenia, ALT and AST increased (two patients each). Six patients experienced a total of 11 SAEs. Of these, five events were considered related to the study treatment (febrile neutropenia [*n* = 2], neurotoxicity, respiratory arrest and cardiac arrest [each *n* = 1]). Febrile neutropenia resolved after 1 and 2 days for each case, and the neurological event was resolved 3 days after the initial event. Two days after the initial SAE, the patient who had respiratory and cardiac arrests had a third cardiac arrest which had a fatal outcome.

**Table 4:** Treatment-related AEs occurring in > 1 patient in AFM11-102

Adverse event	Cohort 1 (*n* = 2)	Cohort 2 (*n* = 1)	Cohort 3 (*n* = 3)	Cohort 4 (*n* = 3)	Cohort 5 (*n* = 5)	Cohort 6 (*n* = 3)	Overall *n* = 17
Patients with any TEAE related to study treatment	2	1	1	3	4	2	13
Pyrexia	0	1	1	2	2	1	7
Alanine aminotransferase increased	0	0	0	0	2	1	3
Aspartate aminotransferase increased	0	0	0	0	2	1	3
Tremor	0	0	0	1	1	1	3
Bone pain	0	0	0	0	2	0	2
Febrile neutropenia	1	0	0	1	0	0	2
Headache	0	0	1	0	0	1	2
Myalgia	0	0	1	0	1	0	2
Neutropenia	0	0	0	1	1	0	2

*AE *adverse event; *TEAE *treatment-emergent adverse event

Dose of AFM11 by cohort (cycle 1 week 1, cycle 1 week 2 onward) was as follows: cohort 1, 0.0007 μg/kg/week, 0.002 μg/kg/week; cohort 2, 0.002 μg/kg/week, 0.006 μg/kg/week; cohort 3, 0.007 μg/kg/week, 0.02 μg/kg/week; cohort 4, 0.02 μg/kg/week, 0.06 μg/kg/week; cohort 5, 0.06 μg/kg/week, 0.18 μg/kg/week; cohort 6, 0.13 μg/kg/week, 0.4 μg/kg/week

TEAEs leading to the withdrawal of the study drug occurred in two patients; of these events, two SAEs of grade 4 respiratory arrest and cardiac arrest in the same patient were considered related to the study drug. A total of four patients died during the study; of these, two patients died due to progressive disease and two patients died due to a TEAE (one event each of actinomycotic pulmonary infection and septic shock), neither event was considered related to the study treatment.

There were no relevant changes in haematology, chemistry parameters or vital signs.

#### Efficacy

Overall, 14 patients had a bone marrow assessment and were assessed for efficacy. An objective response was reported in three patients (one patient in each of cohorts 4, 5 and 6), with a complete response in two patients and a complete response with incomplete recovery of haematological counts in one patient. Refractory disease and progressive disease were reported in four and seven patients, respectively. One patient from cohort 5 achieved a negative minimal residual disease status in the blood at cycle 2 and cycle 3.

At the time of data cut-off, 16 patients had died; median overall survival was 53 days (95% Cl: 35.0, 124.0) and the median 6-month overall survival rate was 17.6% (95% Cl: 4.3%, 38.3%).

#### Pharmacokinetics

Blood samples for measurement of AFM11 serum concentrations were obtained from all 17 patients. Adequate serum concentrations for PK analysis were only available for six patients; data are presented in Table S3.

#### Pharmacodynamics and Immunogenicity

Overall, there were reductions in CD3 + , CD4 + , CD8 + , CD19 + and CD20 + lymphocytes, with a median percent decrease from baseline of 92.2% in CD19 + cells and 91.1% in CD20 + cells at day 8 of cycle 1 and of 89.3%, 92.9% and 83.8% in CD3 + , CD4 + and CD8 + cells, respectively, at cycle 1 day 2. One patient with 6.2 cells/μL CD19 and 6.0 cells/μL CD20 at baseline experienced total B-cell aplasia (0 CD19 and CD20 cells/μL that was evident at cycle 1, day 8 [prior to dose increase] and the cycle 1 evaluation visit).

There were no clinically meaningful trends in change from baseline in IFN-γ, IL-2, IL-6, IL-10 or TNF-α. Overall, 4/17 patients had a positive ADA titre at the cycle 1 evaluation visit; however, this was pre-existing in all patients pre-dose in cycle 1 (baseline). Therefore, no patients developed ADAs while on treatment with AFM11.

## Discussion

These studies aimed to evaluate the safety, PK and activity of AFM11 in two patient populations. There was no evidence of clinical activity in patients with NHL, but biological activity was demonstrated in patients with ALL. Reductions in B-cell populations seen in AFM11-102 are consistent with the expected mode of action of AFM11 and demonstrate that patients received a biologically active dose of AFM11.

In contrast to the observations with other T-cell therapies, neither an increase in cytokines nor cytokine release syndrome was noted in patients receiving AFM11. Although the MTD was not formally established either in patients with NHL or in those with ALL, the MTD is likely to have been exceeded as two patients experienced a DLT in the highest dose groups in each study (0.03 μg/kg for patients with NHL and 0.13 μg/kg/week cycle 1 week 1 and 0.4 μg/kg/week cycle 1 week 2 onwards for patients with ALL).

It is also acknowledged that neurological side effects are pronounced adverse effects of CD19-targeting T-cell therapies such as blinatumomab, axicabtagene ciloleucil and tisagenlecleucel [13, 19, 20]. While it is unclear why AFM11 may differ to other T-cell therapies, these limitations suggest alternative mechanisms of engaging the immune system may be warranted to provide a more tolerable treatment. Activating the innate immune system to direct NK cell-mediated cytotoxicity and macrophage-mediated phagocytosis as well as priming of adaptive immune responses may prove to be an effective and well-tolerated approach [21, 22].

At present, several bispecific antibodies that engage T cells or NK cells to enhance the innate immune response are in development, including those being investigated for the treatment of CD30 + lymphomas. This includes a bispecific, tetravalent chimeric antibody construct, AFM13, specifically targets CD30 on Hodgkin’s lymphoma (HL) cells and recruits and activates NK cells by binding to CD16A. In a phase 1 study in patients with R/R HL, AFM13 treatment was well tolerated, and AEs were typically mild or moderate in severity [23]. In addition, when AFM13 was investigated in combination with an anti-PD-1 therapy in patients with R/R HL, it was generally well tolerated with a safety profile comparable to AFM13 or anti-PD-1 monotherapy [24]. In preclinical studies, the innate cell engager approach, targeting CD16A and thereby innate immunity, has been found to result in significantly lower levels of cytokine release than T-cell engagers [25]. Having two high-affinity anti-CD16A domains means that binding to NK cells is bivalent, preventing cross-linking to neighbouring NK cells that could lead to off-target NK cell activation and potential cytokine release [25].

In conclusion, AFM11 treatment was associated with frequent neurological adverse reactions that were severe in some patients. In ALL, some signs of activity, albeit short-lived, were observed whereas no activity was observed in patients with NHL; therefore, further clinical development was terminated.

## Supplementary Information

**Additional file 1: Figure S1.** AFM11-101 study flow diagram. **Figure S2.** AFM11-102 study flow diagram. **Table S1.** Baseline characteristics per cohort in the AFM11-101 study. **Table S2.** Baseline characteristics per cohort in the AFM11-102 study. **Table S3.** PK parameters of AFM11 following two weekly intravenous infusions of AFM11 in study AFM11-102.

## Data Availability

The authors declare that the data supporting the findings of this study are available within the article.

## Abbreviations

ADA: Antidrug antibody
AE: Adverse event
ALL: Acute lymphoblastic leukaemia
ALT: Alanine aminotransferase
AST: Aspartate aminotransferase
CNS: Central nervous system
CT: Computed tomography
CTCAE: Common Terminology Criteria for Adverse Events
DLT: Dose-limiting toxicity
ECOG: Eastern Cooperative Oncology Group
HL: Hodgkin lymphoma
IFN: Interferon
IL: Interleukin
MTD: Maximum tolerated dose
NHL: Non-Hodgkin lymphoma
NK: Natural killer
PK: Pharmacokinetic
R/R: Relapsed and/or refractory
SAE: Serious adverse event
SD: Standard deviation
TEAE: Treatment-emergent adverse event

## References

[1] Wild C, Weiderpass E, Stewart B, editors. World cancer report: cancer research for cancer prevention. Lyon: International Agency for Research on Cancer; 2020. http://publications.iarc.fr/586. 39432694

[2] Chao MP (2013). Treatment challenges in the management of relapsed or refractory non-Hodgkin's lymphoma - novel and emerging therapies. Cancer Manag Res.

[3] National Institutes of Health. National Cancer Institute, Surveillance, Epidemiology, and End Results (SEER) Program. Cancer Stat Facts: Leukemia – Acute Lymphocytic Leukemia (ALL). https://seer.cancer.gov/statfacts/html/alyl.html.

[4] Fielding AK, Richards SM, Chopra R, Lazarus HM, Litzow MR, Buck G (2007). Outcome of 609 adults after relapse of acute lymphoblastic leukemia (ALL); an MRC UKALL12/ECOG 2993 study. Blood.

[5] Gökbuget N, Dombret H, Ribera JM, Fielding AK, Advani A, Bassan R (2016). International reference analysis of outcomes in adults with B-precursor Ph-negative relapsed/refractory acute lymphoblastic leukemia. Haematologica.

[6] Samra B, Jabbour E, Ravandi F, Kantarjian H, Short NJ (2020). Evolving therapy of adult acute lymphoblastic leukemia: state-of-the-art treatment and future directions. J Hematol Oncol.

[7] Tedder TF (2009). CD19: a promising B cell target for rheumatoid arthritis. Nat Rev Rheumatol.

[8] Hammer O (2012). CD19 as an attractive target for antibody-based therapy. MAbs.

[9] Reusch U, Duell J, Ellwanger K, Herbrecht C, Knackmuss SH, Fucek I (2015). A tetravalent bispecific TandAb (CD19/CD3), AFM11, efficiently recruits T cells for the potent lysis of CD19(+) tumor cells. MAbs.

[10] Wang K, Wei G, Liu D (2012). CD19: a biomarker for B cell development, lymphoma diagnosis and therapy. Exp Hematol Oncol.

[11] Raufi A, Ebrahim AS, Al-Katib A (2013). Targeting CD19 in B-cell lymphoma: emerging role of SAR3419. Cancer Manag Res.

[12] Kantarjian HM, Lioure B, Kim SK, Atallah E, Leguay T, Kelly K (2016). A Phase II Study of Coltuximab Ravtansine (SAR3419) monotherapy in patients with relapsed or refractory acute lymphoblastic leukemia. Clin Lymphoma Myeloma Leuk.

[13] Amgen. Blincyto prescribing information. 2020. Available at https://www.pi.amgen.com/~/media/amgen/repositorysites/pi-amgen-com/blincyto/blincyto_pi_hcp_english.pdf.

[14] Kantarjian H, Stein A, Gökbuget N, Fielding AK, Schuh AC, Ribera JM (2017). Blinatumomab versus chemotherapy for advanced acute lymphoblastic leukemia. N Engl J Med.

[15] Simon R, Freidlin B, Rubinstein L, Arbuck SG, Collins J, Christian MC (1997). Accelerated titration designs for phase I clinical trials in oncology. J Natl Cancer Inst.

[16] Cheson BD, Pfistner B, Juweid ME, Gascoyne RD, Specht L, Horning SJ (2007). Revised response criteria for malignant lymphoma. J Clin Oncol.

[17] Nagorsen D, Kufer P, Baeuerle PA, Bargou R (2012). Blinatumomab: a historical perspective. Pharmacol Ther.

[18] Topp MS, Gökbuget N, Stein AS, Zugmaier G, O'Brien S, Bargou RC (2015). Safety and activity of blinatumomab for adult patients with relapsed or refractory B-precursor acute lymphoblastic leukaemia: a multicentre, single-arm, phase 2 study. Lancet Oncol.

[19] Novartis (2020). Kymriah prescribing information.

[20] Kite Pharma (2021). Yescarta prescribing information.

[21] Demaria O, Cornen S, Daëron M, Morel Y, Medzhitov R, Vivier E (2019). Harnessing innate immunity in cancer therapy. Nature.

[22] Schuster IS, Coudert JD, Andoniou CE, Degli-Esposti MA (2016). "Natural regulators": NK cells as modulators of T cell immunity. Front Immunol.

[23] Rothe A, Sasse S, Topp MS, Eichenauer DA, Hummel H, Reiners KS (2015). A phase 1 study of the bispecific anti-CD30/CD16A antibody construct AFM13 in patients with relapsed or refractory Hodgkin lymphoma. Blood.

[24] Bartlett NL, Herrera AF, Domingo-Domenech E, Mehta A, Forero-Torres A, Garcia-Sanz R (2020). A phase 1b study of AFM13 in combination with pembrolizumab in patients with relapsed or refractory Hodgkin lymphoma. Blood.

[25] Ellwanger K, Reusch U, Fucek I, Wingert S, Ross T, Müller T (2019). Redirected optimized cell killing (ROCK®): a highly versatile multispecific fit-for-purpose antibody platform for engaging innate immunity. MAbs.

