# Supplementary material for: Safety of AFM11 in the treatment of patients with B-cell malignancies: findings from two phase 1 studies
Source: Trials. 2023 Jan 3;24:4. doi: 10.1186/s13063-022-06982-7 (PMC9808944; doi:10.1186/s13063-022-06982-7)
Supplement: Supplementary file 1 — Additional file 1: Figure S1. AFM11-101 study flow diagram. Figure S2. AFM11-102 study flow diagram. Table S1. Baseline characteristics per cohort in the AFM11-101 study. Table S2. Baseline characteristics per cohort in the AFM11-102 study. Table S3. PK parameters of AFM11 following two weekly intravenous infusions of AFM11 in study AFM11-102. [file 13063_2022_6982_MOESM1_ESM.docx]

**Supplementary Figures and Tables**

Figure S1: AFM11-101 study flow diagram


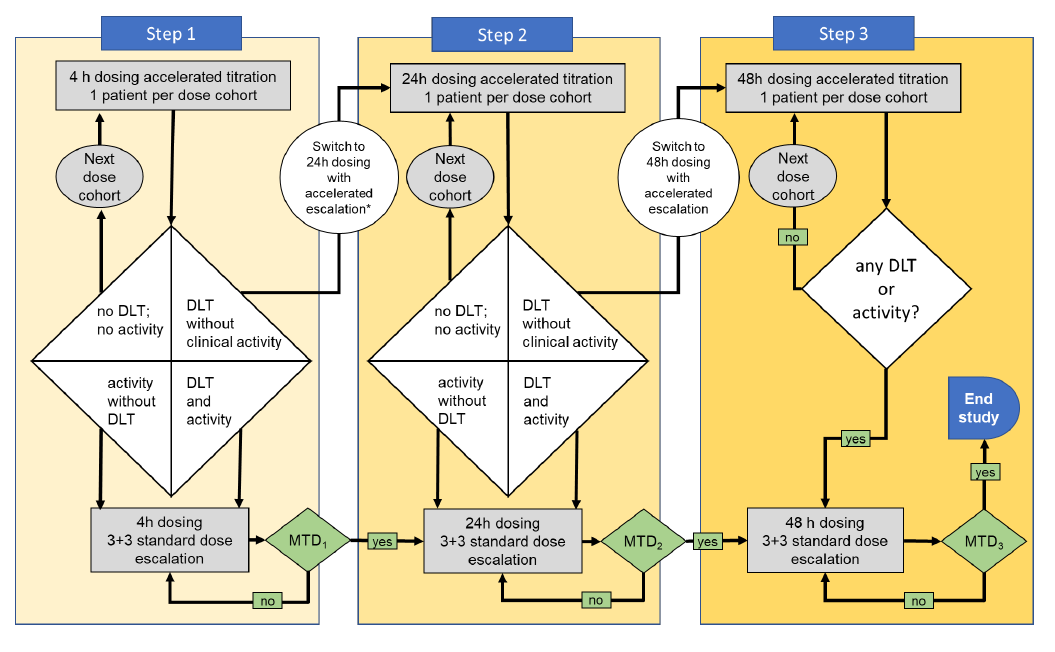


*Only in the first 4 dose cohorts
DLT, dose limiting toxicity; MTD, maximum tolerated dose.

Figure S2: AFM11-102 study flow diagram


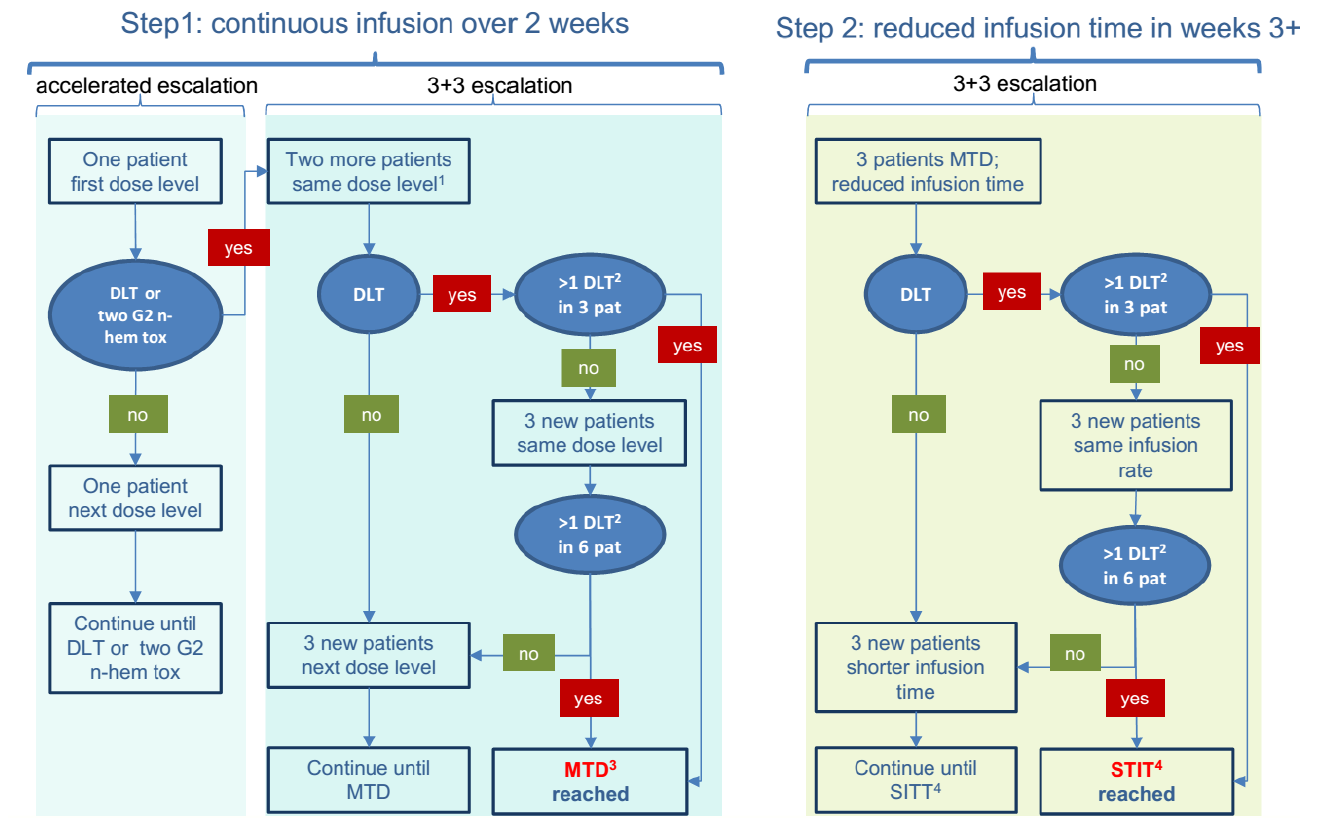


^1^In case a DLT occurs during accelerated titration, 5 additional patients will be enrolled to the same dose level to make it a cohort of 6 patients; ^2^See exception with respect to neurotoxicity; ^3^If the MTD is exceeded at a given dose level and only 3 patients have been enrolled into the previous dose level, an additional 3 patients will be recruited to this dose level to ensure a total of 6 patients have been treated at the MTD; ^4^STIT, shortest tolerated infusion time. Same rules apply as in Note 3.
DLT, dose limiting toxicity; G2, grade 2; MTD, maximum tolerated dose; n-hem tox, non-haematological toxicity; pat, patient; STIT, shortest tolerated infusion time.

Table S1: Baseline characteristics per cohort in the AFM11-101 study

|  | **AFM11 dose level (µg/kg)** | | | | | | |  |
| --- | --- | --- | --- | --- | --- | --- | --- | --- |
|  | Cohort 1  0.0003  (n=1) | Cohort 2  0.001  (n=8) | Cohort 3  0.003  (n=1) | Cohort 4  0.003  (n=1) | Cohort 5  0.003  (n=1) | Cohort 6  0.01  (n=2) | Cohort 7  0.03  (n=2) | Overall  (n=16) |
| Age (years), mean (SD) | 69.0  (NA) | 59.3  (12.36) | 59.0  (NA) | 64.0  (NA) | 74.0  (NA) | 39.5  (NA) | 42.5 (30.41) | 56.5  (16.52) |
| Median  (min, max) | 69.0  (NA) | 59.5  (46, 78) | 59  (NA) | 64  (NA) | 74  (NA) | 39.5  (23, 56) | 42.5  (21, 64) | 61.5 (21, 78) |
| Sex (male, %) | 1 (100) | 4 (50) | 1 (100) | 0 | 1 (100) | 2 (100) | 1 (50) | 10 (62.5) |
| Race, n (%)  White  Asian | 1 (100)  0 | 7 (87.5)  1 (12.5) | 1 (100)  0 | 1 (100)  0 | 1 (100)  0 | 2 (100)  0 | 2 (100)  0 | 15 (93.8)  1 (6.2) |
| ECOG PS, n (%)  Grade 0  Grade 1  Grade 2 | 1 (100)  0  0 | 2 (25)  6 (75)  0 | 0  1 (100)  0 | 1 (100)  0  0 | 0  1 (100)  0 | 1 (50)  1 (50)  0 | 1 (50)  1 (50)  0 | 6 (37.5)  10 (62.5)  0 |
| Diagnosis, n (%)  DLBCL  TBCL  Follicular lymphoma  Marginal zone lymphoma  Mantel cell lymphoma  Other | 1 (100)  0  0  0  0  0 | 2 (25)  2 (25)  2 (25)  1 (12.5)  1 (12.5)  0 | 1 (100)  0  0  0  0  0 | 1 (100)  0  0  0  0  0 | 0  0  1 (100)  0  0  0 | 1 (50)  0  0  0  0  1 (50) | 1 (50)  0  0  0  0  1 (50) | 7 (43.8)  2 (12.5)  3 (18.8)  1 (6.3)  1 (6.3)  2 (12.5) |
| Principal stage at screening, n (%)  Stage I  Stage II  Stage III  Stage IV | 0  1 (100)  0  0 | 1 (12.5)  0  2 (25)  5 (62.5) | 0  0  0  1 (100) | 0  0  0  1 (100) | 0  0  0  1 (100) | 0  0  0  2 (100) | 0  0  0  2 (100) | 1 (6.3)  1 (6.3)  2 (12.5)  12 (75) |
| Prior lines of treatment, n (%)  2  3  4  >5 | 1 (100)  0  0  0 | 1 (12.5)  2 (25)  2 (25)  3 (37.5) | 0  0  0  1 (100) | 0  0  1 (100)  0 | 1 (100)  0  0  0 | 1 (50)  0  0  1 (50) | 0  1 (50)  1 (50)  0 | 4 (25)  3 (18.8)  4 (25)  5 (31.3) |

DLBCL, diffuse large B-cell lymphoma; ECOG, Eastern Cooperative Oncology Group; NA, not applicable; PS, performance status; SD, standard deviation TBCL, T cell/B cell lymphoma.

Table S2: Baseline characteristics per cohort in the AFM11-102 study

|  | Cohort 1  (n=2) | Cohort 2  (n=1) | Cohort 3  (n=3) | Cohort 4  (n=3) | Cohort 5  (n=5) | Cohort 6  (n=3) | Overall  (n=17) |
| --- | --- | --- | --- | --- | --- | --- | --- |
| Age (years), mean (SD) | 46.5 (19.09) | 47.0  (NA) | 51.3 (22.85) | 65.0 (2.65) | 25.6 (6.88) | 59.7 (11.06) | 46.8 (18.80) |
| Median  (min, max) | 47  (33, 60) | 47  (NA) | 63  (25, 66) | 66  (62, 67) | 22  (19, 36) | 61  (48, 70) | 48  (19, 70) |
| Sex, male, n (%) | 2 (100) | 0 | 0 | 1 (33) | 3 (60) | 1 (33) | 7 (41.2) |
| Race, white, n (%) | 2 (100) | 1 (100) | 3 (100) | 3 (100) | 5 (100) | 3 (100) | 17 (100) |
| ECOG PS, n (%)  Grade 0  Grade 1  Grade 2 | 0  1 (50)  1 (50) | 0  1 (100)  0 | 0  3 (100)  0 | 1 (33)  2 (67)  0 | 1 (20)  4 (80)  0 | 1 (33)  2 (67)  0 | 3 (17.6)  13 (76.5)  1 (5.9) |

ECOG, Eastern Cooperative Oncology Group; NA, not applicable; PS, performance status; SD, standard deviation.

Table S3: PK parameters of AFM11 following two weekly intravenous infusions of AFM11 in study AFM11-102

|  | Patient number | | | | | | | | | | | |
| --- | --- | --- | --- | --- | --- | --- | --- | --- | --- | --- | --- | --- |
|  | 1 | | 2 | | 3 | | 4 | | 5 | | 6 | |
|  | Est | CV^†^ | Est | CV^†^ | Est | CV^†^ | Est | CV^†^ | Est | CV^†^ | Est | CV^†^ |
| CL (mL/h) | 269 | 6.80 | 2450 | 18.5 | 868 | 11.6 | 3960 | 25.2 | 5310 | 6.23 | 5980 | 12.4 |
| t_1/2_ (h) | 12.0 | 14.3 | 2.97 | 49.8 | 22.1 | 30.5 | 1.52 | 58.4 | 7.88 | 15.1 | 2.38 | 27.7 |
| V_ss_ (mL) | 4680 | 16.7 | 10500 | 52.9 | 27600 | 30.6 | 8660 | 65.8 | 60400 | 16.8 | 20600 | 30.6 |
| AUC* (pg.h/mL) | 7780 | | 2970 | | 11200 | | 5910 | | 8100 | | 3130 | |

*Average concentration at steady state; CV^†^, Coefficient of variation of individual parameter.
AUC, area under the concentration curve; CL, clearance; CV, coefficient of variation; Est, estimate; t1/2, half-life; Vss, apparent volume of distribution.
